# Deformability based Cell Sorting using Microfluidic Ratchets Enabling Phenotypic Separation of Leukocytes Directly from Whole Blood

**DOI:** 10.1038/s41598-017-06865-x

**Published:** 2017-07-26

**Authors:** Quan Guo, Simon P. Duffy, Kerryn Matthews, Emel Islamzada, Hongshen Ma

**Affiliations:** 1 Department of Mechanical Engineering, University of British Columbia, 2054-6250 Applied Science Lane, Vancouver, BC V6T 1Z4 Canada; 2 Department of Urologic Science, University of British Columbia, Vancouver, BC Canada; 3 Vancouver Prostate Centre, Vancouver General Hospital, Vancouver, BC Canada

## Abstract

The separation of leukocytes from whole blood is a prerequisite for many biological assays. Traditional methods require significant sample volumes and are often undesirable because they expose leukocytes to harsh physical or chemical treatment. Existing microfluidic approaches can work with smaller volumes, but lack selectivity. In particular, the selectivity of microfluidic systems based on microfiltration is limited by fouling due to clogging. Here, we developed a method to separate leukocytes from whole blood using the microfluidic ratchet mechanism, which filters the blood sample using a matrix of micrometer-scale tapered constrictions. Deforming single cells through such constrictions requires directionally asymmetrical forces, which enables oscillatory flow to create a ratcheting transport that depends on cell size and deformability. Simultaneously, oscillatory flow continuously agitates the cells to limit the contact time with the filter microstructure to prevent adsorption and clogging. We show this device is capable of isolating leukocytes from whole blood with 100% purity (i.e. no contaminant erythrocytes) and <2% leukocytes loss. We further demonstrate the potential to phenotypically sort leukocytes to enrich for granulocytes and lymphocytes subpopulations. Together, this process provides a sensitive method to isolate and sort leukocytes directly from whole blood based on their biophysical properties.

## Introduction

Leukocytes, or white blood cells (WBCs), can provide access to a veritable treasure-trove of information on the health status of each individual. For example, leukocytes can serve as indicators of pathogen infection^1^, especially for leukocyte-specific pathogens such as the human immunodeficiency virus (HIV). Additionally, during T-cell adoptive therapy isolated leukocytes can be expanded *ex*-*vivo* for re-targeting and re-infusion into an individual^2^. An important prerequisite for many leukocyte assays is the depletion of contaminating erythrocytes, or red blood cells (RBCs), as well as the isolation of specific phenotypes. Established methods are typically based on centrifugation, fluorescence-activated^3, 4^, and magnetic-activated cell sorting^5–7^. These methods are often costly, require relatively large samples, and expose leukocytes to harsh chemical and physical treatment, which may cause cell lysis, contribute to undesirable phenotypic changes^8^, and corrupt the information obtained from these cells^9^.

Recently, a number of microfluidic methods have been developed for phenotypic separation of leukocytes from small volumes of whole blood. Leukocytes may be separated based on size and deformability using hydrodynamic chromatography, such as deterministic lateral displacement (DLD) or margination. In DLD, cells navigate a 2D array of obstacles along different streamlines based on their distinct size and deformability^10–12^. Margination relies on preferential migration of leukocytes along a vessel wall and clustering of RBCs in the middle of a vessel, resulting from the hydrodynamic lift generated through the Fåhræus-Lindqvist effect^13–16^. Alternatively, leukocytes may be separated based on distinct dielectric properties, which arise from cell size as well as nuclear and membrane morphology^17, 18^, and magnetophoresis, which depletes deoxygenated RBCs based on their specific magnetic susceptibility^19–21^. A significant challenge for separating leukocytes from whole blood using these approaches is the lack of selectivity both in the depletion of RBCs, as well as in the selectivity for specific phenotypes.

A potentially simple and selective approach to isolate leukocytes from whole blood is microfiltration. Since RBCs can deform through openings (1–2 µm) much smaller than their diameter (~8 µm), while circulating leukocytes are spherical with diameters 6–8 μm^22^, microfilters consisting of pillars^23, 24^, weirs^23, 25, 26^, and membrane pores^23, 27^ have been developed to separate these leukocytes from RBCs. A key limitation in directly processing whole blood using microfiltration is the potential for cells to clog and foul the filter microstructures. Clogging of the filter matrix alters its hydrodynamic resistance and the filtration force on each cell, resulting in a significant reduction in selectivity within the filters. Cross flow filtration can be employed to divert trapped cells using a secondary flow tangential to the filter surface^23, 28–31^ but does not completely release the cells adsorbed within the filter microstructures and significantly reduces the overall selectivity as well as throughput.

In this study, we employed the microfluidic ratchet mechanism to perform perpetual microfiltration of leukocytes from whole blood. Building on results from cell deformability studies performed using AFM^32^, micropipette aspiration^33, 34^ and its microfluidic derivative^35^, we hypothesize that erythrocytes, leukocytes and leukocyte subpopulations can be sensitively separated based on their distinct cell deformability. Previously, the microfluidic ratchet mechanism has been shown to be able to effectively separate viable circulating tumor cells from whole blood^36^ with significant improvements in yield over conventional methods as well as to separate erythrocytes infected with *Plasmodium falciparum* ^37^ from uninfected erythrocytes to improve the sensitivity of malaria diagnosis. Using oscillating flow of cells through an array of funnel shaped microstructures, we show that whole blood can be processed without clogging or fouling the filter matrix. Consequently, this process enables highly selective separation of leukocytes and erythrocytes, as well as demonstrates the potential for phenotypic sorting of leukocytes.

## Results

### Microfluidic Ratchets for Continuous Blood Cells Sorting based on Deformability

**Figure 1:**
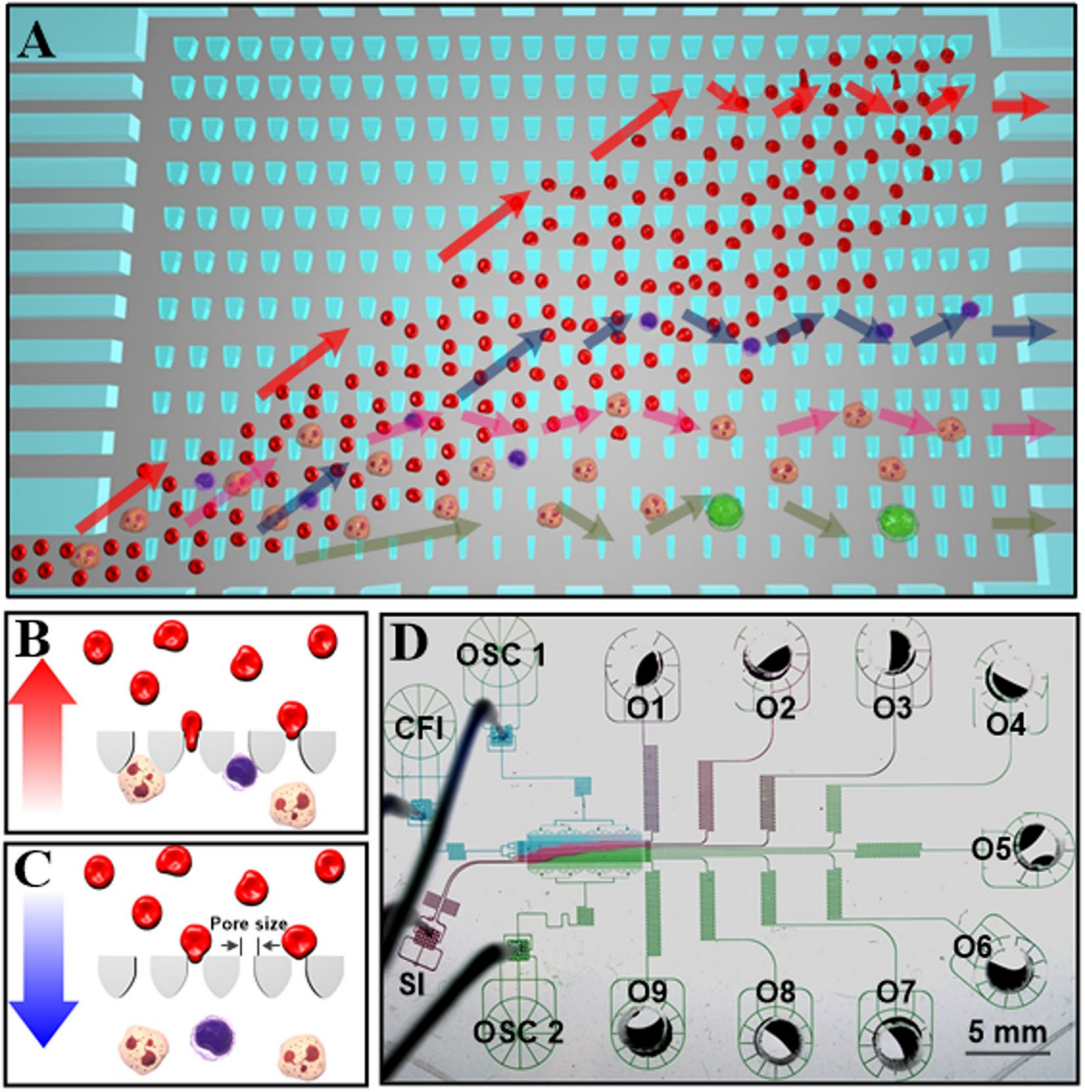
Principle and design of the microfluidic ratchet cell sorting device. (**A**) Schematic illustration of the cell sorting region consisting of a 2D array of funnel microstructures with progressively smaller pore sizes from the bottom to the top row. Unprocessed whole blood is introduced at the bottom-left corner of the array while cells flow along a diagonal path under a combined biased oscillatory flow and cross flow. Smaller and softer RBCs flow into the outlets lining the top of the sorting region while the flow of leukocytes is restricted based on their size and deformability, which divert each cell into specific outlets. Colored arrows indicate the ratchet motions of erythrocytes and different subsets of leukocytes. (**B**,**C**) The underlying principle of the microfluidic ratchet involves selectively transporting cells using a ratcheting effect. Smaller and more deformable cells flow across tapered constrictions during forward flow and are prevented from returning during reverse flow. Larger and less deformable cells are trapped by the constriction and released with each reverse flow. (**D**) Image of the microfluidic device infused with different food color dyes illustrating components of the microfluidic device including the sorting region, sample inlet (SI), cross-flow inlet (CFI), oscillatory flow inlets (OSC1 and OSC2), and nine outlets (O1–O9).

The microfluidic ratchet mechanism filters cells through a funnel-shaped micro-pillar array with minimum gap widths (or pore sizes) that gradually decrease from the bottom row to the top row of the array (Fig. 1A). The sample cells are infused at the bottom-left corner of the matrix, and transported through the array using a vertical oscillatory flow, at the same time as a constant horizontal cross flow. These flows combine to propel the cells in a zigzag diagonal path through the constriction matrix with each oscillation cycle, until they reach a limiting pore size that prevents further upward transport (Fig. 1A). Upon reaching this point, the cells oscillate between two funnel rows, allowing the horizontal flow to divert them toward a specific outlet.

The operation of this cell sorting mechanism relies on two key design principles: First, cells are deformed through an asymmetrical, funnel-shaped constriction, which we previously showed to require less pressure to transit cells in the direction of the taper (Fig. 1B) than against the taper (Fig. 1C)^38^. Second, cells are transported using a biased oscillatory flow that results in a net upward migration. The coupling of these two effects enables selective transport of cells based on their ability to deform through microscopic constrictions in a ratcheting manner. The ability to filter cells using oscillatory flow is essential to the perpetuation of the filtration process, as each reverse flow clears the funnel filters of non-specific adherent cells to eliminate clogging, which enables the processing of high-density samples like whole blood. Moreover, since cells come into contact with the funnel filters only momentarily during oscillatory flow, the hydrodynamic resistance of the filter, and consequently the filtration force experienced by each incoming cell, remains consistent during the entire process (supplemental information, Figure S1, Table S1). Finally, unlike traditional binary separation, the array-based sorting process allows a heterogeneous sample to be sorted into multiple fractions in a single run.

### Microfluidic Device Design

The fractionation of the cell sample into the outlets after sorting is determined by the dimensions and arrangement of the funnel pores within the sorting matrix. The funnel array consists of 35 rows and 630 columns, occupying an overall area of 1875 × 9525 µm. The funnel pore size is kept constant for each set of four rows and progressively decreases with each row from 8 µm at the bottom to 2 µm at the top of the sorting region. The gap between each row of funnels is 50 µm. As a result, the 35 rows of funnel array sort the input whole blood into 9 fractions in outlet 1 to 9 (O1–O9), with O1 corresponding to the most deformable fraction and O9 corresponding to the least deformable fraction. This geometry of the funnel constrictions is designed to deform cells laterally through 8, 6.5, 5.5, 4.5, 4, 3.5, 3, 2.5 and 2 µm pore sizes, while providing stress relief in the orthogonal direction, which is kept constant at a thickness of 11–15 µm. Fluid flow in the sorting matrix is manipulated through supporting microchannel networks, including a blood sample inlet (SI), cross-flow inlet (CFI) and oscillation flow inlets (OSC1 and OSC2), as illustrated in Fig. 1D. The design of the microchannel networks follows a hydrodynamic resistance model, which determines the filtration forces applied across the funnel filter and the flow rate inside the sorting matrix. A detailed hydrodynamic model of fluid flow in this microstructure is presented in the supplemental materials. Based on this prototype design, the microfluidic ratchets are able to process whole blood at a rate of 5 µl per hour. While this throughput is low compared to other biophysical cell separation methods^10–12, 14, 15^, the throughput of this device could ultimately be improved by increasing the filtration area, as well as by paralleling the mechanism.

### Separation of Leukocytes from Erythrocytes

**Figure 2:**
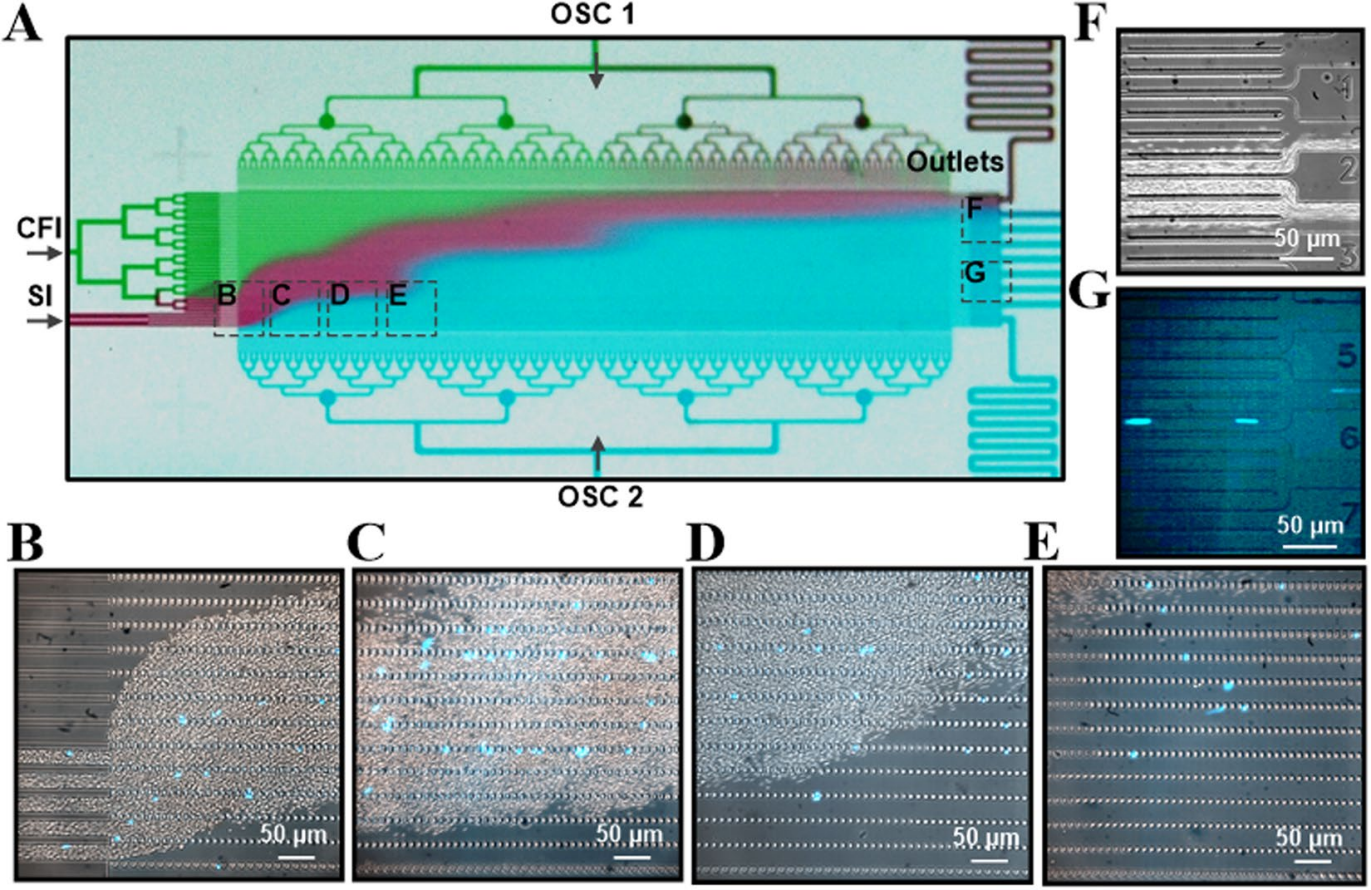
Microfluidic ratchets to separate leukocytes directly from whole blood. (**A**) Image of the sorting region of microfluidic ratchets infused with food color dyes illustrating the diagonal trajectory of the sample inlet flow. (**B–E**) Images of the leukocytes stained with Hoechst 33342 being separated from the whole blood and traversing diagonally through the funnel matrix. (**F**,**G**) Bright field and fluorescence images of the outlets showing the separate reservoirs into which RBCs and leukocytes are collected.

Initially, the device was evaluated of its ability to separate leukocytes from the highly abundant erythrocytes. To distinguish leukocytes from erythrocytes, blood samples were pre-stained with Hoechst 33342 DNA stain and infused into the sorting region (Fig. 2A). As shown in Fig. 2B–E, the sample formed the characteristic diagonal trajectory. Individual leukocytes (blue) that have reached their limiting funnel constrictions were found to transit horizontally as expected (Fig. 2E and supplemental video). As a result, RBCs were collected in fractions O1-O3 (Fig. 2F; 2, 2.5 and 3 µm pores) while leukocytes were preferentially segregated among outlets O4-O9 (Fig. 2G; 3.5, 4, 4.5, 5.5, 6.5 and 8 µm pores). These results indicated that 3 µm is a suitable cut-off pore size for the separation of erythrocytes and leukocytes, which is similar to 3.5 µm pores used by other microfilters with pillar, weir, and membrane configurations^23^. It is important to note that staining the leukocytes required washing the cells, which removed the plasma component of blood. In this case, the blood cells were re-suspended at ~45% hematocrit. Both stained blood cells and unstained whole blood were tested and observed to behave equivalently.

**Figure 3:**
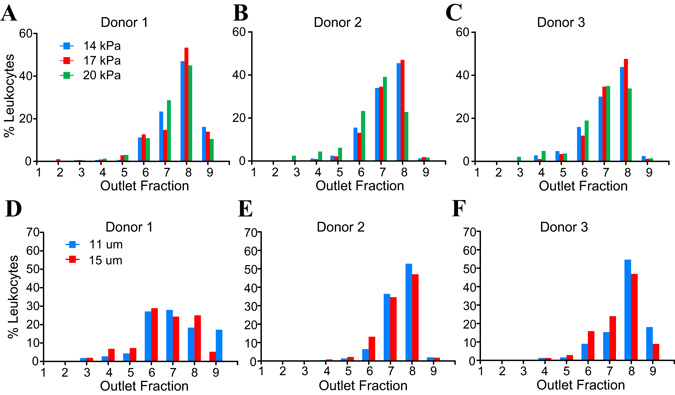
Leukocyte distribution at outlets as a function of filtration pressure applied at OSC2 and funnel thickness. (**A–C**) Leukocyte deformability profiles of three different donors at various filtration pressure with fixed funnel thickness of 11 µm. (**D–F**) Leukocyte deformability profiles of three donors at two different funnel thickness at a fixed filtration pressure of 17 kPa.

**Figure 4:**
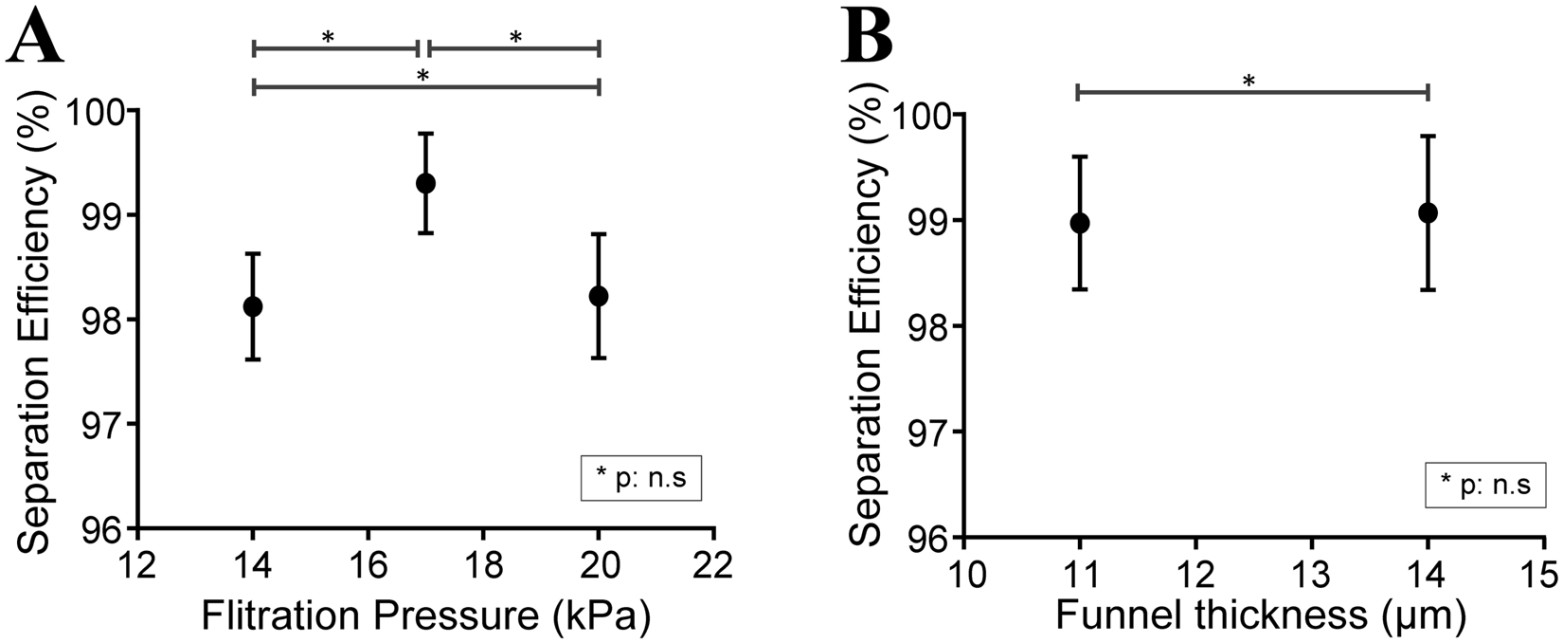
Performance of deformability-based separation of leukocytes from whole blood as a function of (**A**) filtration pressure and (**B**) funnel thickness. (P values determined using Student’s T-test).

The separation of leukocyte from erythrocytes was investigated as a function of oscillation pressure and funnel thickness, which varied from 14–20 kPa and 11–15 µm respectively. This operating range was established using previous experiments of acceptable operating conditions (see supplemental information). As shown in Fig. 3A–C, higher filtration pressures increased the frequency for cells to transit narrower openings, represented by a slight leftward shift of leukocytes’ outlet distributions for three different donors’ blood samples. Increasing the funnel thickness from 11 to 15 µm allows each cell to have greater vertical extension during deformation. However, as show in Fig. 3D–F, this change has minimal effect on the leukocyte distributions. RBCs were found exclusively in O1–3 in all scenarios except when the filtration pressure was decreased to 14 kPa, in which case, a small fraction of RBCs escaped into O4, leading to decreased leukocyte separation efficiency. Here, separation efficiency is defined as the pure fraction of leukocytes captured in outlets over the total number of leukocytes entering the sorting region (Fig. 4A). Since device thickness (11–15 µm) does not seem to have a strong influence on the efficiency of leukocytes separation from RBCs (Fig. 4B), we select a modest oscillation pressure of 17 kPa, as well as a device thickness of 11 µm to make the device robust to potential debris in the sample. Based on this configuration the microfluidic ratchet device is able to obtain a leukocyte separation efficiency of 98–99% (Fig. 4).

### Separation of granulocytes and lymphocytes subpopulations from whole blood

**Figure 5:**
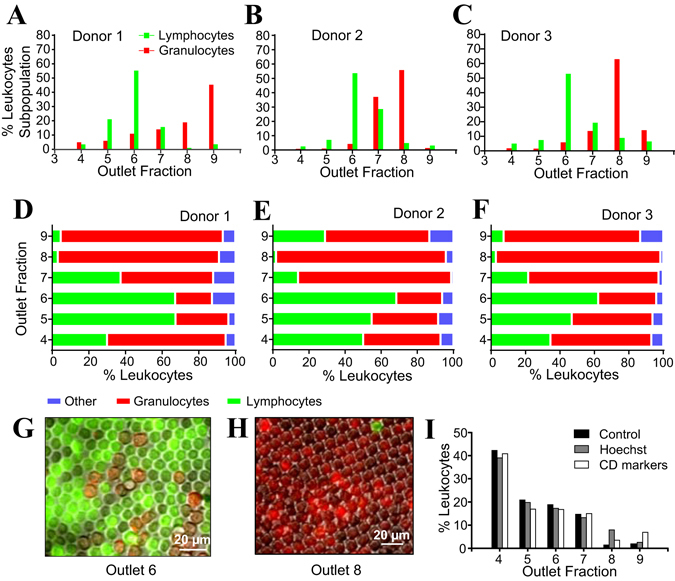
Separation of lymphocytes and granulocytes from whole blood. (**A**–**C**) Deformability profiles of lymphocytes and granulocytes enriched from blood of three different donors. (**D**–**F**) Relative abundance of lymphocytes and granulocytes in each outlet. (**G**,**H**) Images of the immunostained lymphocytes (green) in O6 and granulocytes (red) in O8. (**I**) Comparison of distributions of leukocytes in O4–9 of three different samples: (1) whole blood without stain, (2) with Hoechst 33342 and (3) with anti-CD markers conjugated with fluorescence tags.

To investigate whether deformability based sorting could separate granulocyte and lymphocyte subpopulations directly from whole blood, we pre-stained donor blood for CD3 and CD19 in order to identify the majority of lymphocytes (T cells and B cells) as well as for CD66b to identify granulocytes. Following microfluidic ratchet enrichment, leukocytes were distributed among O4–O9. Lymphocytes were preferentially distributed around O6 (retained between 4.5 to 5.5 µm pore size), where such cells can be obtained at 62–68% purity (Fig. 5A–F). Granulocytes were distributed around outlets O8 or O9 (retained between 6.5 to 8 µm funnel opening), where such cells can be obtained at 88–95% purity in these outlets (Fig. 5A–F). The images in Fig. 5G and H demonstrates that lymphocytes and granulocytes comprise the majority of the cells in O6 and O8 respectively. Based on their distribution into outlets, it was observed that leukocytes experience significantly less deformation than would be expected within smaller capillaries, such as pulmonary capillaries, which has circular constrictions that are ~5 µm in diameter^39, 40^. Specifically, lymphocytes (average diameter 7 µm) are typically blocked by the 4.5 × 11 µm constriction while neutrophils (average diameter 7.5 µm) are blocked by the 6.5 × 11 µm constriction.

To investigate whether the fluorescence stains or the staining process alters leukocyte deformability to affect their distribution after sorting, experiments were performed to compare the outlet distributions of whole blood samples without stain, with Hoechst 33342 stain, as well as with fluorescently conjugated markers using samples from the same donor. As shown in Fig. 5I, the leukocyte distribution is very consistent, which confirms that fluorescence staining has no effect on leukocyte deformability.

### Intra- and inter-individual differences of outlet distributions of leukocytes and their subsets

**Figure 6:**
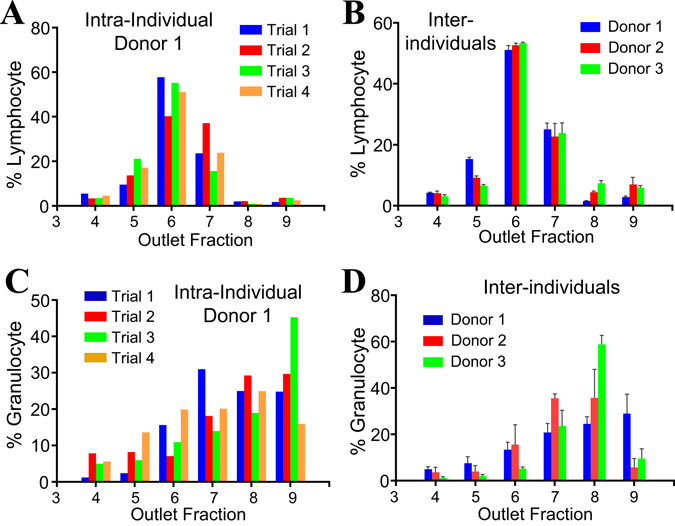
Outlet distributions of lymphocytes and granulocytes. (**A**,**B**) Outlet distributions of lymphocytes from three donors. (**C**,**D**) Outlet distributions of granulocytes from three donors. Each inter-individual distribution profile data point is a mean of triplicate experiments. The error bars in Fig. 6B and D represent the standard deviation of results from 3 trials.

We investigated the intra- and inter-individual variability of granulocyte and lymphocyte distributions after sorting. The intra-individual variability for lymphocytes and granulocytes were established through four independent experiments on blood from a single donor, while the inter-individual variability was assessed using experiments on blood from three different donors. Both intra- and inter-individual distributions for lymphocytes were remarkably consistent, with ~60% retention in O6 (Fig. 6A–B). In contrast, granulocyte distribution varied significantly, both intra- and inter-individually (Fig. 6C–D). This result is not surprising as granulocytes are dominated by neutrophils, which represent more than 90% of these cells, and neutrophils are known to adopt different biophysical characteristics upon activation by chemical^40^ or mechanical stresses^41^.

### Outlet distributions of monocytes

**Figure 7:**
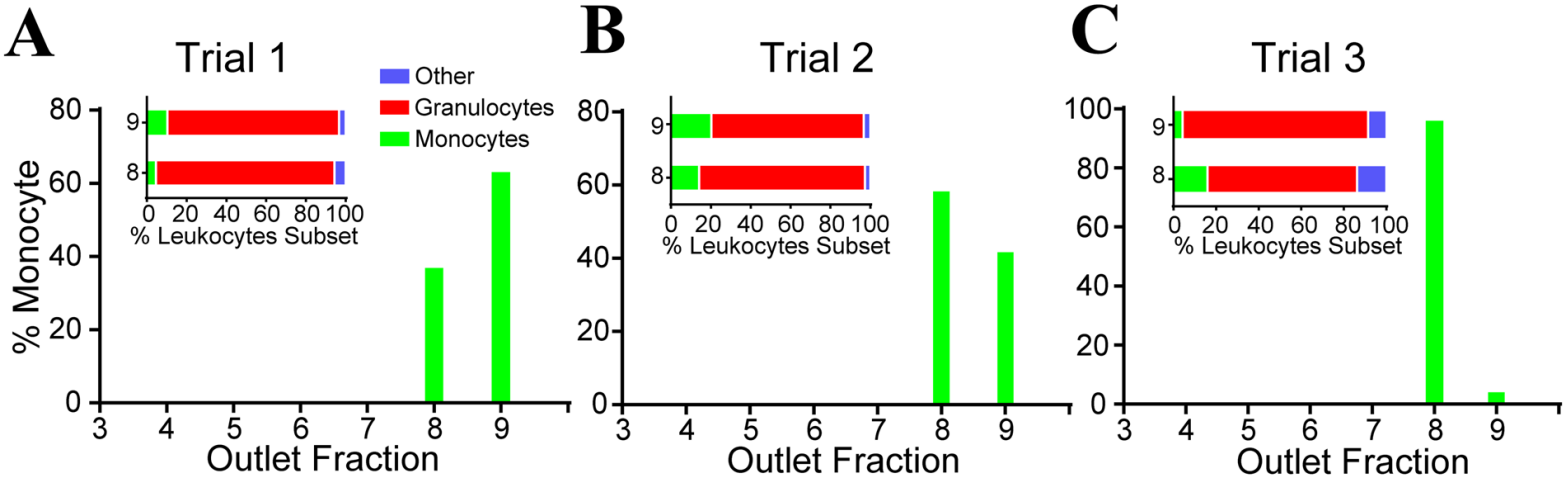
Distribution of monocytes sorted from whole blood from the same donor in three trials. Inset graphs show the composition of leukocytes subsets in O8 and 9.

Lastly, the distribution of monocytes was evaluated to determine whether they could be separated from lymphocytes and granulocytes, based on deformability. Monocytes make up 2–8% of the total leukocyte population. They average 7.5 µm in diameter^22^, making them slightly larger than either lymphocytes (6.2 µm) or granulocytes (7.0–7.3 µm). We sorted monocytes from whole blood after pre-staining with anti-CD14 marker and observed that they were retained primarily in outlets O8–O9, by the 6.5 µm constrictions (Fig. 7). This result suggests that these cells could be efficiently enriched from lymphocytes but not from granulocytes using the current version of the microfluidic ratchets device. The purity of monocytes in O8 range 4.5–20%, relative to granulocytes, which represents only a modest increase from the standard frequency of these cells in peripheral blood leukocyte population. However, the nearly exclusive accumulation of monocytes within these outlets suggests that future iterations of this device could resolve monocytes from granulocytes by optimization of the pore size (in the range between 6.5 and 8 µm) and funnel thickness.

## Discussion

The microfluidic ratchet mechanism employed in this study overcomes some key limitations of both conventional microfiltration and microfluidic biophysical cell sorting systems to enable leukocyte separation from whole blood with 100% purity, as well as phenotypic sorting of leukocyte subpopulations. Microfiltration methods developed previously using pillars^23, 24^, weir^23, 25, 26^ and membrane pores^23, 27^ have all shown a tendency to clog, which increases hydrodynamic resistance, reduces selectivity and necessitates increased filtration pressure or periodic washing of the filter region. While secondary tangential flow partly alleviates device clogging^23, 28–31^, the oscillating flow used in microfluidic ratchet sorting enables clog-free and nearly perpetual processing of high-density whole blood samples. Since the deformation of cells is less than that which leukocytes normally experience when traversing through micro-capillaries in the body^39, 40^, this process does not adversely affect the viability of the leukocytes.

The performance of the microfluidic ratchet mechanism can be compared to other filtration methods using several metrics, including separation efficiency (the fraction of input leukocytes captured), leukocyte purity (the proportion of leukocytes in the output cell suspension), RBC depletion rate and overall sample throughput. In comparison with microfiltration, the key advantages of microfluidic ratchet sorting are the separation efficiency and leukocyte purity. While previous microfiltration methods typically report poor separation efficiency or fail to report this metric (Table 1), microfluidic ratchet consistently generates pure leukocyte isolates from whole blood with only 1–2% loss. The microfluidic ratchet also compares favorably with other biophysical separation (Table 2), including magnetophoresis, dielectrophoresis, and leukocyte margination. Magnetophoresis uses paramagnetic properties of RBCs to deplete these cells from the leukocyte suspension^19–21^. While RBC depletion using this approach exceeds 95%, the separated leukocytes remain at low relative purity because of the large number of starting RBCs (10^6^/µl) in whole blood. Conversely, size-based sorting strategies, such as dielectrophoresis^17^, DLD^10–12^ and leukocyte margination^14–16^ can achieve superior leukocyte separation efficiency. However, a key advantage of the microfluidic ratchet mechanism is that it combines both size and deformability based selection to not only generate pure leukocyte suspensions but also to segregate leukocyte subpopulations, despite significant overlap in the sizes of different leukocyte subpopulations^22^.

**Table 1:** Performances of recent research in leukocytes separation from whole blood using microfiltration.

Filtration Strategies and Filter Types*	Performance Metrics				
	Leukocyte Separation Efficiency	Leukocyte Purity	RBC Depletion Rate	Throughput	Requirement for Blood dilution
Microfluidic Ratchet *Current Study*	98–99%	100%	100%	5 µL hour^−1^	No
Dead end; Pillar^46^	18–25%	—(<1%)	84–89%	15–50 µL min^−1^	Yes (1:2 in PBS)
Dead end; weir^26^	71%	~10% (210-fold enrichment)	n.d.	4000 cells s^−1^	Yes (1:10 in PBS)
Dead end; weir^47^	60%	n.d.	n.d.	3–15 µL hour^−1^	No
Dead end; pore^27^	>90%	n.d.	n.d.	Only process 1.5 µL whole blood	No
Dead end; pore^23^	72–85%	n.d.	n.d.	Only process 200 µL whole blood	No
Dead end; weir^23^	~70%	n.d.	n.d.	Only process <50 µL whole blood	No
Dead end; pillar^23^	70–95%	n.d.	n.d.	Only process 300 µL whole blood	No
Cross flow; weir^29^	98%	72%	n.d.	3.6 µL hour^−1^	Yes
Cross flow; pore^31^	27.4 ± 4.9%	93.5 ± 0.5%	n.d.	~17 µL min^−1^	No
Cross flow; pillar ^30^	90%	<1%	90%	10 µL min^−1^	No
Cross flow; weir^30^	20%	<1%	40%	10 µL min^−1^	Yes (1:100 in PBS)
Cross flow; pillar^23^	70–95%	n.d.	n.d.	20 µL min^−1^	No
Cross flow; pillar^28^	97%	<1%	50%	5 µL min^−1^	n.d.

n.d. Data not available.

*References in uppercase.

**Table 2:** Performances specifications of recent microfluidic research in leukocytes separation from whole blood using biophysical methods.

Method*	Performance Metrics				
	Leukocyte Separation Efficiency	Leukocyte Purity	RBC Depletion Rate	Throughput	Requirement for Blood Dilution
Microfluidic Ratchet *Current Study*	98–99%	100%	100%	5 µL hour^−1^	No
Magnetophoresis^19^	n.d.	n.d	93.5%	5 µL hour^−1^	Yes (1:10 in PBS)
Magnetophoresis^20^	n.d.	n.d	95%	0.5–0.7 mL hour^−1^	Yes (1:20 in PBS)
Magnetophoresis^21^	n.d.	n.d	93.7%	0.12–0.92 µL min^−1^	Yes (1:40 in PBS)
Dielectrophoresis (DEP)^17^	76.9–92.1%	n.d.	n.d.	50 µL hour^−1^	Yes (1:5 in PBS)
DLD^10^	99%	n.d	n.d.	1 µL min^−1^	No
Leukocyte margination^14^	80%	90%	n.d.	600 µL hour^−1^	Yes (0.5–2% hematocrit)
Leukocyte margination^15^	97%	100%	n.d.	1 µL min^−1^	Yes (1:1000 in PBS)
Leukocyte margination^16^	67%	1%	n.d.	1 µL hour^−1^	No

n.d. Data not available.

*References in uppercase.

A key disadvantage of the microfluidic ratchet mechanism is sample throughput, which at 5 µl per hour for the prototype studied here is significantly less than other methods, such as DLD, which is capable of inhibiting clot formation^42^ and processing 10 ml of whole blood per minute^12^. This limited throughput results from the ratchet transport process requiring oscillatory flow, which significantly improved selectivity, but at the cost of throughput. The throughput of the prototype device studied here is sufficient for many leukocyte assays, including flow cytometry, ELISA, and chemotaxis. However, we also recognize there are many applications where greater throughput is desired. In these situations, throughput can be increased by parallelizing the mechanism, as well as optimizing the device design for the specific application, as we have done for a device to enrich for circulating tumor cells, which has a sample throughput of 1 ml/hour^36^.

In summary, the microfluidic ratchet mechanism enables highly selective sorting of leukocytes directly from whole blood. We showed this mechanism can achieve clear separation of lymphocytes and granulocytes, and we believe with further refinement, the separation of other leukocyte phenotypes can be achieved. Another important application of this technology is the interrogation of the phenotypic plasticity of leukocytes to identify biomechanical changes associated with leukocyte activation, such as neutrophil activation during cancer progression^43^ and viral infection^44^. This approach represents a potential alternative to flow cytometry to enrich for leukocytes that adopt specific activation states. After sorting, the fractionated cells are immediately available for downstream molecular characterization, such as immunofluorescence, transcriptome analysis, or cytokine secretion assays. Together, the microfluidic ratchet mechanism represents a simple and compelling approach for biophysical separation and characterization of leukocytes as part of biological assays.

## Methods

### Microfluidic fabrication

#### Photolithography

The microfluidic ratchet device consists of a single fluidic layer fabricated using soft-lithography of polydimethylsiloxane (PDMS) silicone. The mold for the microstructures consists of two photo-lithographically defined layers fabricated on a silicon wafer. The sorting region containing a matrix of funnels was fabricated using SU-8 3010 photoresist (MicroChem, Newton, MA, USA) producing features with thickness of 11–15 µm. The supporting microfluidic channels (SI, OSC1, OSC2, CFI and Outlets) were made from SU-8 3025 photoresist with a thickness of ~25 µm. The patterns for both masks were drawn using AutoCAD software.

The SU-8 3010 microstructures were fabricated on a cleaned 100 mm silicon wafer. After dehydration baking at 200 °C for 5 minutes, photoresist SU-8 3010 was spread onto the wafer at 1500 to 1800 rpm for 30 seconds to create a thickness of 11 µm to 15 µm, as measured using a profilometer (Alpha step 200). The wafer was then soft baked at 95 °C for 4 minutes before being exposed to UV light in a mask aligner with uniform exposure for 40 seconds. The exposed wafer was given a post exposure bake at 65 °C for 1 minute, 95 °C for 3 minutes and then 65 °C for 1 minute. Finally, the wafer was developed using SU-8 developer (MicroChem). The geometry of the SU-8 3010 photoresist was stabilized by further baking with ramped temperature at the acceleration of 100 °C hour^−1^ from 40 °C to 200 °C, held at 200 °C for one hour, and then gradually cooled to 40 °C.

Next, the SU-8 3025 microstructures were added to the SU-8 3010 silicon wafer. SU-8 3025 photoresist was spin-coated on the wafer at 4500 rpm for 30 seconds. The coated wafer was soft baked at 65 °C for 1 minute, 95 °C for 4.5 minutes, and then 65 °C for 1 minute. The designed mask for the SU-8 3025 pattern was then aligned with the SU-8 3010 pattern and exposed for 55 seconds. After waiting for approximately 30 minutes, the wafer was developed using SU-8 developer (MicroChem). The finished structure was measured to be 18–20 µm in thickness.

#### Soft-lithography

Polyurethane-based plastic (Smooth-Cast ONYX SLOW, Smooth-On) was used to make replicas of the microstructures on the master silicon wafer, as previously described^45^. PDMS microfluidic devices were then fabricated from these molds using soft-lithography of RTV 615 PDMS (Momentive Performance Materials).

After baking, the cured microfluidic device was removed from its mold, and holes for the fluidic introduction ports including cross flow inlet (CFI) and sample inlet (SI) as well as the oscillation inlets (OSC1 and OSC2), were punched into it using a 0.5 mm outer diameter hole punch (Technical Innovations, Angleton, TX, USA). The outlets were punched using a 3 or 4 mm diameter puncher. The microfluidic device was then bonded by oxygen plasma (Model PDC-001, Harrick Plasma) to a blank layer of PDMS previously spin-coated onto a blank silicon wafer at 1500 rpm for 1 minute and then subsequently bonded to a standard microscope slide (50 × 75 mm, Fisher Scientific).

### Blood sample collection

Blood from healthy donors was obtained via venipuncture in 6 ml EDTA vacutainer tubes (BD, Canada) following informed consent. The approval was obtained from the University of British Columbia’s Clinical Research Ethics Board. The study was carried out in strict accordance with the guidelines and regulations of the University of British Columbia’s Clinical Research Ethics Board.

### DNA staining of leukocytes

Whole blood (200 µl) was stained with Hoechst 33342 (Sigma Aldrich) to label the DNA of leukocytes. Hoechst (5 µg ml^−1^) was added at 1:100 (v/v) to the sample and incubated for 30 minutes at room temperature in the dark. Subsequently, the sample was washed three times in Phosphate Buffered Saline (PBS; CaCl_2_-free and MgSO4-free; Invitrogen) with 2% heat-inactivated fetal bovine serum (FBS). After the last wash, the supernatant was replaced with PBS containing 0.2% Pluronic^TM^ F-127 (Invitrogen) at 1:1 (v/v) ratio with the packed blood cells to obtain the similar hematocrit as whole blood.

### Leukocyte Immunophenotyping

Lymphocytes were stained with Alexa Fluor® 488 Mouse Anti-Human CD3, clone SP34-2 (557705, BD) and Alexa Fluor® 488 Mouse Anti-Human CD19, clone HIB19 (557697, BD). Granulocytes were stained with Alexa Fluor® 647 Mouse Anti-Human CD66b, clone G10F5 (561645, BD) and monocytes with Alexa Fluor® 488 Mouse Anti-Human CD14, clone M5E2 (557700, BD), all according to manufacturer’s instructions. After staining and washing, the supernatant was replaced with PBS containing 0.2% Pluronic^TM^ F-127 (Invitrogen) at 1:1 (v/v) ratio with the packed blood cells. Lymphocytes and neutrophils are observed at the outlets through fluorescence microscope. Multichannel images were taken as shown in Fig. 5G and H.

### One sentence summary

We developed a microfluidic device capable of biophysically separating leukocytes directly from whole blood with 100% purity and <2% loss, as well as sorting leukocytes to enrich for granulocytes and lymphocytes.

## Electronic supplementary material

Supplemental Information

Supplemental Video
