# Supplementary material for: Deformability based Cell Sorting using Microfluidic Ratchets Enabling Phenotypic Separation of Leukocytes Directly from Whole Blood
Source: Sci Rep. 2017 Jul 26;7:6627. doi: 10.1038/s41598-017-06865-x (PMC5529452; doi:10.1038/s41598-017-06865-x)
Supplement: Supplementary file 1 — Supplemental Information [file 41598_2017_6865_MOESM1_ESM.pdf]

Supplemental Information for:

Deformability based Cell Sorting using Microfluidic Ratchets Enabling Phenotypic Separation of Leukocytes Directly from Whole Blood

Quan Guo[a], Simon P. Duffy[a], Kerryn Matthews[a], Emel Islamzada[a], and Hongshen Ma[a,b,c*]

a Department of Mechanical Engineering, University of British Columbia, 2054-6250 Applied Science Lane, Vancouver, BC, Canada V6T 1Z4
b Department of Urologic Science, University of British Columbia, Vancouver, BC, Canada
c Vancouver Prostate Centre, Vancouver General Hospital, Vancouver, BC, Canada

*** Corresponding Author:**

Dr. Hongshen Ma

hongma@mech.ubc.ca

Department of Mechanical Engineering
University of British Columbia
2054-6250 Applied Science Lane
Vancouver, BC, Canada V6T 1Z4

ONE SENTENCE SUMMARY:

We developed a microfluidic device capable of biophysically separating leukocytes directly from whole blood with 100% purity and <2% loss, as well as sorting leukocytes to enrich for granulocytes and lymphocytes.

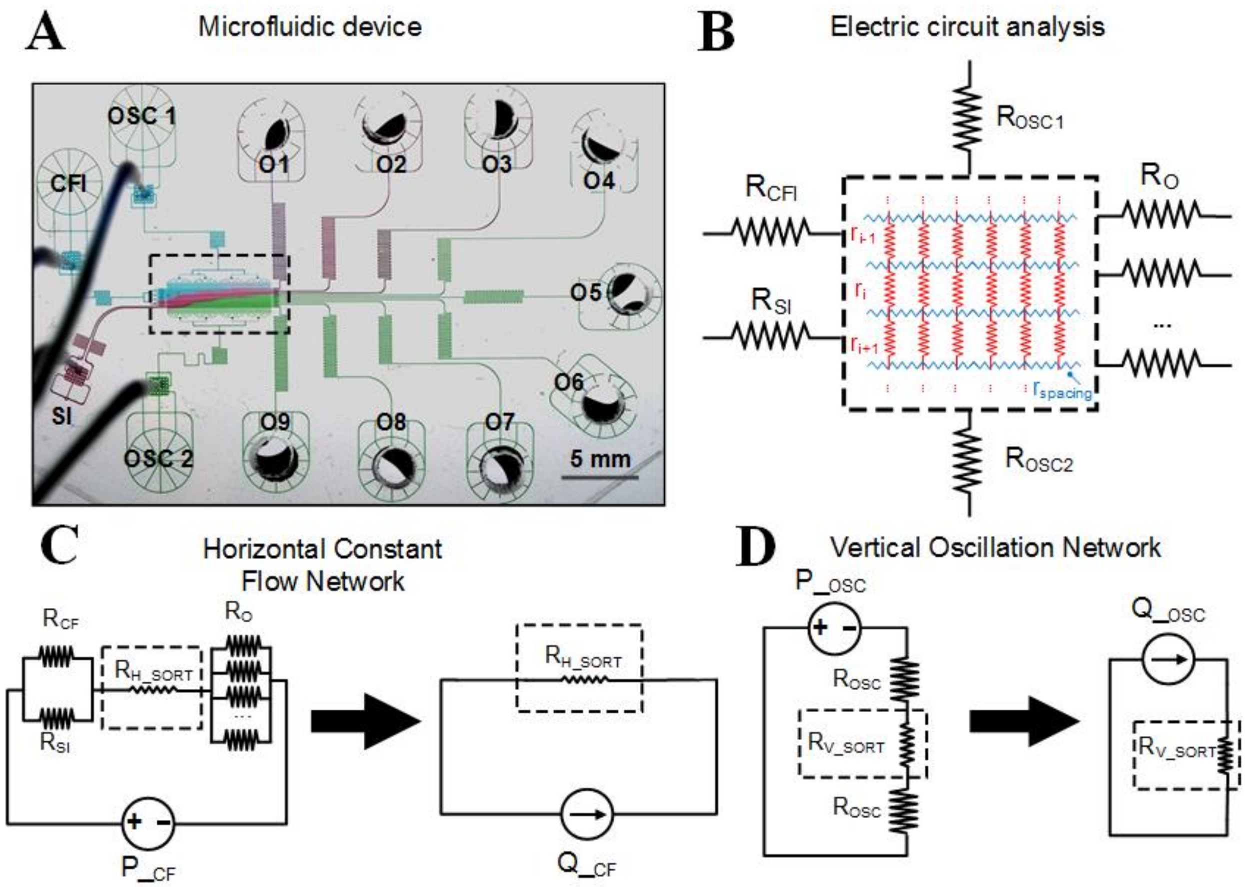

Figure S1: *Hydrodynamic resistance model of the microfluidic device.* ***(A)*** *Image of the microfluidic device and* ***(B)*** *its equivalent hydrodynamic circuit model analysis;* ***(C)*** *electric circuit equivalent to the* ***(C)*** *horizontal flow network and* ***(D)*** *vertical oscillation network*

Table S1. Summary of the hydrodynamic resistance of various components of the device

	Components	Hydrodynamic Resistance ($Pa{\cdot}s{\cdot}m^3$)
**Vertical Oscillatory Flow Channel**	R_{V_SORT}	$2.35{\times}10^{13}$
	R_{OSC}	$6.75{\times}10^{14}$
**Horizontal Flow Channel**	R_{H_SORT}	$7.59{\times}10^{13}$
	R_{SI}	$3.79{\times}10^{14}$
	R_{CFI}	$4.90{\times}10^{14}$
	R_O	$1.54{\times}10^{15}$

Hydrodynamic Model

Laminar fluid flow can be modelled using standard methods of linear electrical circuit analysis. Specifically, fluid flow in a microfluidic channel is linearly proportional to the pressure drop across the length of the channel. For incompressible fluid, the volume is conserved and therefore, pressure and the flow rate can be determined from,

$$\Delta P = R_H \times Q, \quad \text{(S1)}$$

where ΔP is the pressure difference (Pa) across the fluidic channel, Q is the volumetric flow rate ($m^3{\cdot}s^{-1}$) and R_H is the hydrodynamic resistance ($Pa{\cdot}s{\cdot}m^3$). **Figure S1B** illustrates the equivalent hydrodynamic circuit for the leukocyte sorting microfluidic device (**Figure S1A**), which has components including cross flow network (R_{CF}), sample inlet network (R_{SI}), oscillation network (R_{OSC}) and outlet networks (R_O). Fluid flow in the sorting region can be considered as a superposition of the horizontal constant flow circuit (**Figure S1C**) and vertical oscillation flow circuit (**Figure S1D**). In the vertical circuit, the hydrodynamic resistance of the sorting region, R_{V_SORT}, can be determined by adding up the resistances of the individual funnel constrictions,

$$R_{V\_SORT} = \frac{\sum_i^{n_{row}} r_i}{n_{column}}, \quad \text{(S2)}$$

where n_{row} and n_{column} are the number of funnel rows and columns in the matrix, r_i is the resistance of the individual funnel and the value of each r_i is determined using finite element simulation (COMSOL multiphysics). In the horizontal circuit, the hydrodynamic resistance of the sorting matrix, R_{H_SORT}, can be determined from the resistance of the spacing between each funnel row ($r_{spacing}$) using

$$R_{H\_SORT} = \frac{r_{spacing}}{n_{spacing}} , \quad \text{(S3)}$$

where $n_{spacing}$ is the number of horizontal spacings in the sorting matrix.

The supporting microchannel networks (R_O, R_{OSC}, R_{CF} and R_{SI}) are designed to present a dominant hydrodynamic resistance (>50X) over that of the sorting region, allowing precise control of fluid flow using pressure-driven flow from the inlets. The full listing of the hydrodynamic resistance values of these components are in **Table S1**. This hydrodynamic design provides a constant flow rate in the funnel matrix, whose resistance may vary with the number of cells in the funnel matrix, and thereby ensures that each cell experiences a nearly constant deformation pressure. This design further serves to dramatically reduce the pressure applied at the inlets to derive an attenuated pressure for each cell. Specifically, the oscillation pressures ranging from 14 to 20 kPa applied at the oscillation inlets are reduced to approximately 5 to 30 Pa at funnel constrictions, which was on the same order of pressure magnitude for individual leukocyte and erythrocytes deformation through funnels determined previously [1, 2].

Microfluidic Device Operation

Operation of the microfluidic device involves initially introducing the blood sample under pre-determined pressure settings for the blood sample inlet (SI), cross flow inlet (CFI), downward (OSC1) and upward (OSC2) oscillation as well as oscillation frequency for OSC1 and OSC2. These parameters were empirically determined to produce a characteristic diagonal stream across the rectangular sorting region. The filtration pressure (OSC2) is set by observing the angle of the samples diagonal stream. If the filtration pressure (OSC2) is too low, the cells do not have sufficient time to test each row of funnel and will exit the sorting region prematurely. If the

filtration pressure is too high, RBCs will exit the sorting region through the top oscillation channel. This also increases the likelihood of rupturing leukocytes while being squeezed through their limiting funnel opening, causing cell adsorption and potential clogging of the system. The acceptable range of filtration pressure was determined to be 14 kPa to 20 kPa, which confines the cell sample within the sorting region while allowing cells, particularly leukocytes to reach their limiting funnel opening and to be collected in the corresponding outlets. The oscillation was set to bias upwards (OSC2) to filter the cells for 4 seconds and downwards (OSC1) to de-clog the cells for 1 second. To minimalize the interruption of reverse flow for de-clogging, it was determined that 1 second of downward oscillation at 14 kPa was sufficient to release any cells trapped in the funnel constrictions. Upward oscillation longer than 4 seconds may increase the likelihood of cells being adsorbed in the funnels, which may lead to the eventual clogging of the funnel matrix. The pressure for the SI determines the throughput of the sorting process as a smaller SI pressure will limit the number of cells infused into the sorting region. The CFI pressure creates a constant horizontal flow and plays an important role in forming the characteristic diagonal trajectory. A high CFI pressure will force the cells to traverse through the sorting matrix prematurely and a low CFI pressure will cause the sample infused from SI to travel into the CFI networks during the upwards oscillation. Accordingly, the pressure values for both CFI and SI are set between 50-60 mbar (5 to 6 kPa).

FLOW RATE CALCULATION

Based on the hydrodynamic resistance model and acceptable sample inlet pressure determined empirically, the theoretical volumetric flow rate (Q_{TH}) can be calculated using:

$$Q_{TH} = \frac{P_{SI}}{R_{SI-O}} \ , \qquad \text{(S4)}$$

Where P_{SI} is sample inlet pressure (5 - 6kPa) and R_{SI-O} is total hydrodynamic resistance from sample inlet to outlet. Q_{TH} is calculated to be 2.36 - 2.84 µL/hour. The result has been confirmed by experiment. Specifically, the volumetric flow rate is estimated to be 5 µL/hour using:

$$Q = \frac{Total\ number\ of\ leukocytes\ collected\ at\ outlet\ per\ hour}{Concentration\ of\ leukocyte\ in\ the\ input\ sample} \qquad \text{(S5)}$$

Supplemental Video: Leukocyte isolation from whole blood using the microfluidic ratchets. Blood sample was pre-stained with Hoechst 33342 DNA stain and infused into the sorting region. The cells (erythrocytes and leukocytes) formed the characteristic diagonal trajectory. Individual leukocytes (blue) that have reached their limiting funnel constrictions were found to transit horizontally and collected in O4-9 while erythrocytes were collected in O1-3 exclusively.

Supplemental Video.avi
